# A facultative mutualistic feedback enhances the stability of tropical intertidal seagrass beds

**DOI:** 10.1038/s41598-018-31060-x

**Published:** 2018-08-28

**Authors:** Jimmy de Fouw, Tjisse van der Heide, Jim van Belzen, Laura L. Govers, Mohammed Ahmed Sidi Cheikh, Han Olff, Johan van de Koppel, Jan A. van Gils

**Affiliations:** 1 Department of Aquatic Ecology and Environmental Biology, Institute for Water and Wetland Research, Radboud University Nijmegen, Heyendaalseweg 135, 6525 AJ Nijmegen, The Netherlands; 2 Department of Coastal Systems, NIOZ Royal Netherlands Institute for Sea Research, and Utrecht University, P.O. Box 59, 1790 AB Den Burg, Texel The Netherlands; 3 Groningen Institute for Evolutionary Life Sciences (GELIFES), University of Groningen, P.O. Box 11103, 9700 CC Groningen, The Netherlands; 4 Department of Estuarine and Delta Systems, NIOZ Royal Netherlands Institute for Sea Research, and Utrecht University, P.O. Box 140, NL-4400 AC Yerseke, The Netherlands; 5 Ecosystem Management Research Group, Department of Biology, University of Antwerp, Universiteitsplein 1, 2610 Wilrijk, Belgium

## Abstract

Marine foundation species such as corals, seagrasses, salt marsh plants, and mangrove trees are increasingly found to engage in mutualistic interactions. Because mutualisms by their very nature generate a positive feedback between the species, subtle environmental impacts on one of the species involved may trigger mutualism breakdown, potentially leading to ecosystem regime shifts. Using an empirically parameterized model, we investigate a facultative mutualism between seagrass and lucinid bivalves with endosymbiotic sulfide-oxidizing gill bacteria in a tropical intertidal ecosystem. Model predictions for our system show that, by alleviating the build-up of toxic sulfide, this mutualism maintains an otherwise intrinsically unstable seagrass ecosystem. However, an increase in seagrass mortality above natural levels, due to e.g. desiccation stress, triggers mutualism breakdown. This pushes the system in collapse-and-recovery dynamics (‘slow-fast cycles’) characterized by long-term persistent states of bare and seagrass-dominated, with rapid transitions in between. Model results were consistent with remote sensing analyses that suggest feedback-mediated state shifts induced by desiccation. Overall, our combined theoretical and empirical results illustrate the potential of mutualistic feedbacks to stabilize ecosystems, but also reveal an important drawback as small environmental changes may trigger shifts. We therefore suggest that mutualisms should be considered for marine conservation and restoration of seagrass beds.

## Introduction

Mutualisms form vital ecological interactions in a wide range of ecosystems including coral reefs, seagrass beds, peatlands and forests^1–5^. Indeed, more than 90% of all tropical forest plants depend on mutualistic pollinator and/or seed dispersal interactions for reproduction^6,7^, and about 80% of all land plants are involved in mutualistic mycorrhiza-root partnerships^8^. Mutualisms can be particularly pervasive in coral reefs, salt marshes, mangroves, seagrass beds and deep-sea hydrothermal vents where they may strongly depend on mutualistic interactions^2,9–15^. In these marine ecosystems habitat-structuring foundation species alter the environmental conditions thereby facilitating many other species. However, these foundation species are increasingly found to engage in mutualistic interactions which may enable them to persist an even on a broader range of conditions^14,16–19^. As a large part of the associated marine community typically depends on these foundation species, mutualisms involving foundation species do not only affect the species directly involved but may also have a major impact on ecosystem functioning and stability.

Although mutualism can increase the environmental range limits of the species involved, e.g. by improving stress tolerance of species^1,20,21^, recent studies also suggest an important potentially unavoidable downside. Because mutualistic interactions by their very nature generate a positive feedback mechanism between the species involved^4,22,23^, disruption of this feedback may lead to loss of the foundation species in conditions where in depends on mutualism for survival, potentially triggering a sudden shift in the state of the whole ecosystem^24–26^. Minor environmental changes (e.g. increase in temperature or nutrients) may potentially cause mutualism breakdown^4,17,27^. For example, small climate change-related phenological shifts between plants and their pollinators can cause a mismatch between mutualistic partners^28^, and in coral reef mutualisms, subtle temperature increases have been suggested to cause ‘coral bleaching events’^9,29^. Even though mutualism breakdown is likely to become more common in ecosystems in the face of global change, the role of mutualisms involving foundation species for ecosystem stability and the mechanistic understanding of their disruption remains unclear.

Here, we explore how a previously documented facultative mutualism between seagrasses and lucinid bivalves with endosymbiotic sulfide-oxidizing gill bacteria affects the stability and dynamics of a tropical seagrass ecosystem. By trapping suspended particles from the water layer and stabilizing sediments, seagrasses facilitate their own growth as this improves water clarity^30–32^. As a consequence, however, seagrass beds can also create a negative feedback, because the accumulated organic matter in the sediment is decomposed anaerobically by sulfate-reducing bacteria that produce toxic sulfide as a metabolic end-product^2,33^. However, previous work revealed that seagrasses can create a positive feedback by engaging in a mutualistic interaction with lucinid bivalves and their sulfide-oxidizing, gill-inhabiting bacteria to reduce sulfide stress. In return, the bivalves and their endosymbionts not only profit from sulfide that is indirectly provided by seagrasses due to organic matter trapping, but also from oxygen released by seagrass roots^2,17,33^. Lucinid bivalves are found in high densities in the rhizosphere of seagrasses meadows, especially in the tropics where sulfide production is generally high^2,3^.

In our study system, the tropical marine intertidal flats of Banc d’Arguin in Mauritania (West Africa), a sudden seagrass die-off event occurred in 2011 when low-tide drought stress triggered the failure of the facultative seagrass-lucinid mutualism^17^. Although our earlier work suggests that environmental stress trigger the breakdown of the mutualistic seagrass-lucinid feedback, it has not yet been investigated how the mutualism affects ecosystem stability and whether its breakdown can indeed generate the observed ecosystem dynamics. We constructed a differential equation model with empirically informed parameters to (1) investigate the importance of the mutualistic interaction for seagrass ecosystem stability, and (2) gain insight how environmental stress – low-tide desiccation stress in our case – affects ecosystem resilience. We started by analysing how our model system behaves with and without the facultative mutualistic feedback. Second, we investigated whether increases in environmental stress (e.g. drought) could lead to unstable dynamics and ecosystem collapse. Specifically, we analysed the stability of the model over a range of seagrass mortality settings, which we used as a proxy for low-tide desiccation stress. Finally, we used a potential analysis – a method for detecting feedback-mediated shifts – on both model simulation results and remote sensing satellite data seagrass (NDVI) to link our theoretical results to empirical observations.

## Results

### Model simulations

**Figure 1:**
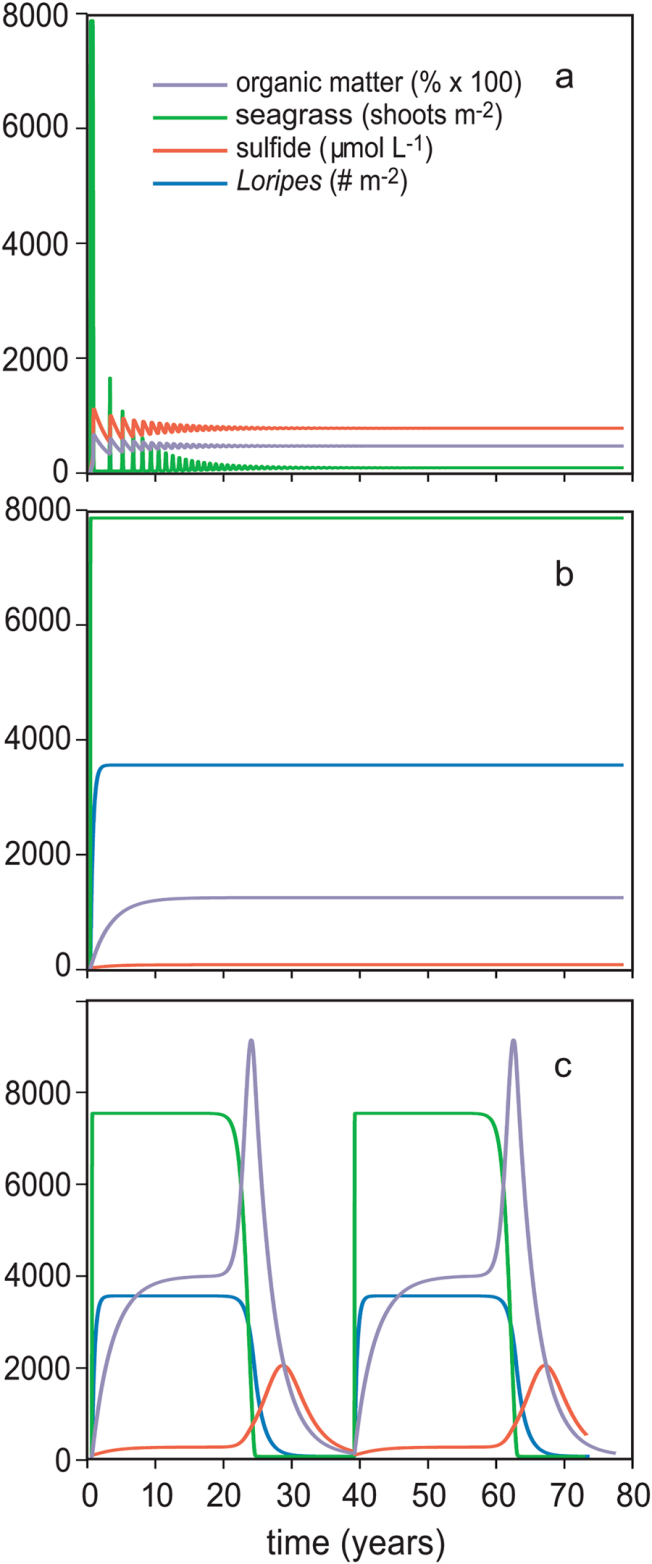
Model simulations of seagrass density *Z* (shoots m^−2^), sulfide concentrations *S* (µmol L^−1^), organic matter *OM* (%) and *Loripes* density *L* (ind. m^−2^). (**A**) Model simulations at default setting without mutualistic interaction displayed damped oscillation (unstable) behaviour due to a negative feedback as a result of organic matter accumulation and sulfide toxicity. (**B**) When adding the mutualistic interaction, any produced sulfide is removed and oscillations disappear. (**C**) An increase (from *m*_*n*_ = 0.007 to *m*_*n*_ = 0.023) of seagrass background mortality causes slow-fast cycles in which the mutualism temporarily buffer against sulfide toxicity, but is not able to remove all sulfide produced, eventually causing seagrass collapse. Recovery occurs when organic matter and sulfide have disappeared from the system.

Model simulations without the mutualism at default settings (Table 1) predict that dense seagrass beds in our study system are not stable. Seagrass shoot densities peak at around 50% of carrying capacity *Z*_*max*_, after which it quickly collapses. This is because seagrass creates a negative feedback on its own growth, by accumulating organic matter that in turn results in enhanced sulfide production. Sulfide toxicity causes the system to collapse, after which a number of damped oscillations follow (Fig. 1a). Incorporation of the mutualistic interaction into the model at default settings yields a stable seagrass bed at carrying capacity. This is possible because the negative effect of seagrass on itself is buffered as toxic sulfide is now removed from the sediment by *Loripes* (Fig. 1b). However, further examination reveals that an increase in seagrass mortality as a simulation of enhanced environmental stress, creates a condition in which *Loripes* is not able to consume all sulfide produced. This causes sulfide to accumulate slowly to the point where it becomes toxic, triggering sudden seagrass degradation. At some point, the die-off then becomes enhanced by the disruption of the mutualistic feedback, sending the system into cyclic collapse and recovery dynamics (Fig. 1c).

**Table 1:** State and parameter values of the models and functions.

Parameters/state variable	Description (unit)	Default value	ref
*Z*	seagrass shoots shoots (m^−2^)	—	
*S*	pore water sulfide concentration (µmol L^−1^)	—	
*OM*	organic matter (%)	—	
*L*	*Loripes *density (ind m^−2^)	—	
*Z*_*max*_	maximum seagrass density (carrying capacity) (shoots m^−2^)	8000	1
*r*	relative growth rate seagrass (day^−1^)	0.35	1–3
*m*_*s*_	maximum seagrass mortality rate by sulfide (day^−1^)	0.5	1,4^a^
*m*_*n*_	natural seagrass mortality rate (day^−1^)	0.007	2
*C*_*om*_	conversion factor relating % OM to sulfide (µmol L^−1^%^−1^ day^−1^)	0.01/ *C*_*z*_	1 ^b^
*C*_*s*_	conversion factor relating loss of sulfide by uptake (ind^−1^ day^−1^ m^2^)	0.0027	1 ^b^
*e*_*s*_	loss of S due to chemical oxidation and loss water layer (day^−1^)	0.29	1
*C*_*z*_	conversion factor for sh/m^2^ to OM% (%shoots^−1^ m^2^)	2.017e^−6^	1
*e*_*m*_	loss of OM due decomposition and export (day^−1^)	0.0009	1
*S*_*min*_	minimum sulfide concentration where toxicity occurs (µmol L^−1^)	200	5
*S*_*max*_	sulfide concentration where the toxicity effect becomes maximal (µmol L^−1^)	1000	5
*r*_*L*_	relative growth rate *Loripes* (ind day^−1^ m^−2^)	26	6 ^a^
*L*_*max*_	maximum *Loripes* density (carrying capacity) (ind m^×2^)	4,900	7
*m*_*L*_	natural *Loripes* mortality rate (day^×1^)	0.002	6 ^a^

^a^value calculated from source; ^b^value dived from experiment (See supplementary Text 1); (1) this study (See supplementary Text 1); (2) van der Heide *et al*.^49^; (3) Peralta *et al*.^66^; (4) Homer and Bondgaard^67^; (5) van der Heide *et al*., Govers *et al*.^2,68^; (6) Ahmedou Salem *et al*.^69^; (7) van der Geest^70^.

**Figure 2:**
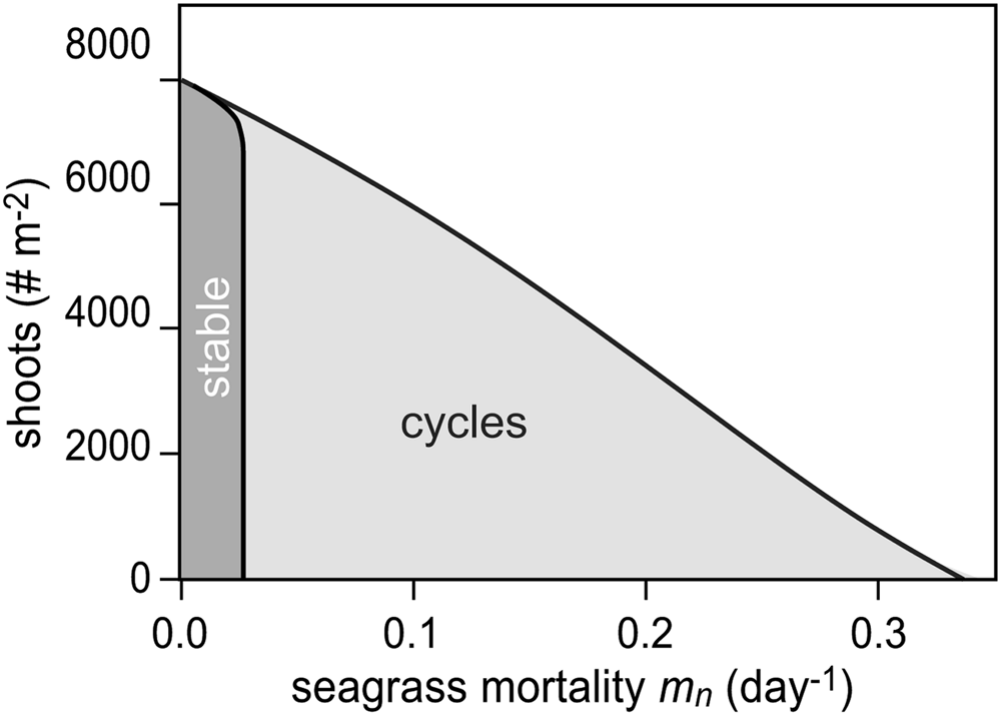
Results of the bifurcation analysis of the model. The system with mutualism is stable at default settings, but displays cyclic behaviour after increase of mortality (*m*_*n*_ > 0.0235).

A more thorough analysis of seagrass mortality *m*_*n*_ in a bifurcation analysis of the model with the mutualism reveals a stable system when seagrass mortality rates remain between 0 and 0.023 day^−1^, but that so-called “slow-fast limit cycles”^34^ occur between 0.023 and 0.35 day^−1^ (Fig. 2). Even though sulfide production eventually outpaces sulfide removal by *Loripes* when *m*_*n*_ > 0.023, collapse of the system is nevertheless dramatically postponed due the buffering effect of sulfide removal by the mutualism. Sensitivity analysis of all other model parameters at otherwise default settings indicate that the default model outcome (i.e. the stabilizing effect of the mutualism) is generally applicable for our study system as large parameter changes (at least 19% change) are required to change the default dynamics of the model (Supplementary Table 2).

### Comparing model and field dynamics

**Figure 3:**
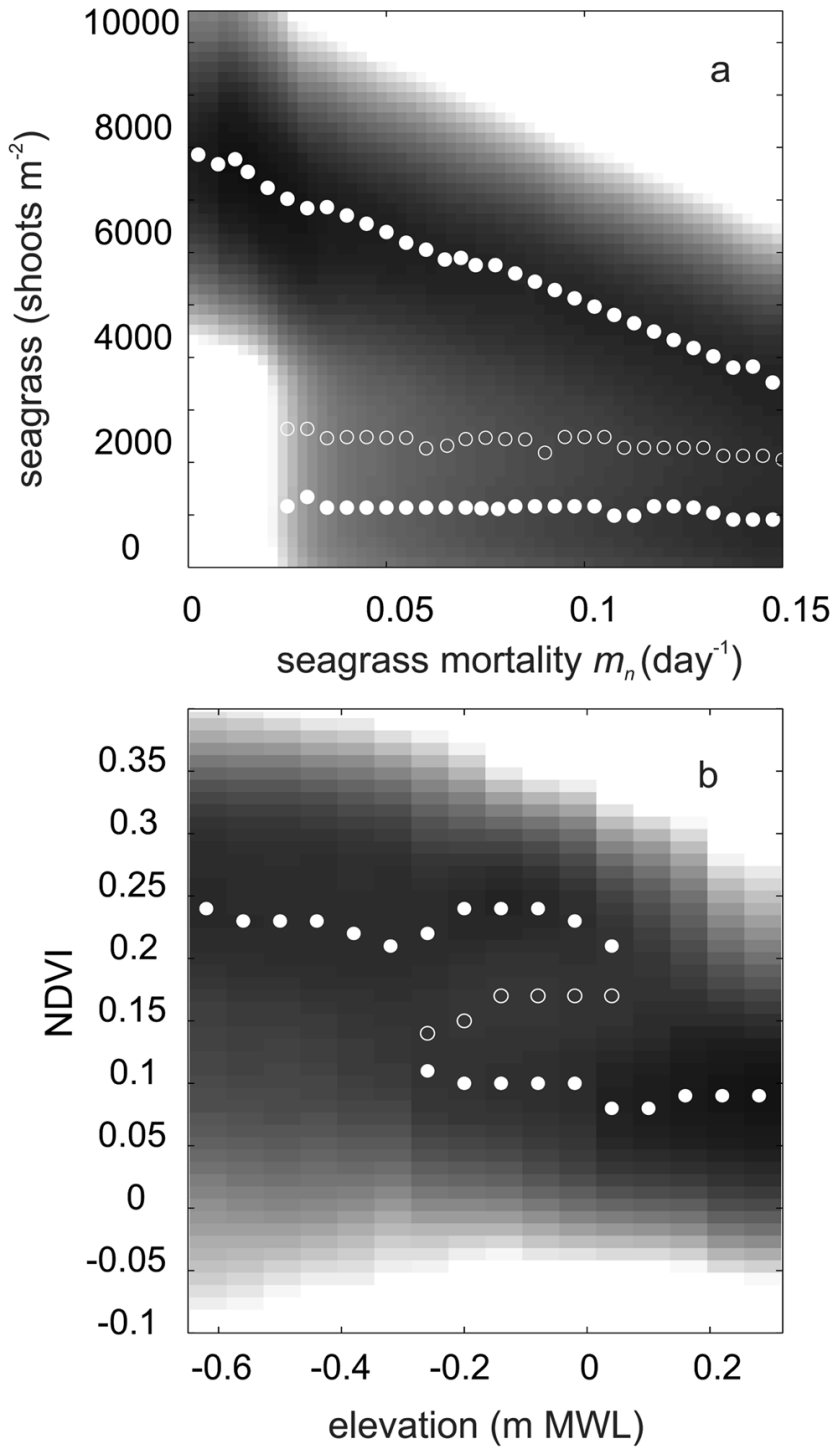
Potential analysis of the simulated seagrass data of the mutualism model and of the NDVI data. Dark and light shades depict shoot density ranges of high and low occurrence, respectively; closed and open markers depict ‘attractors’ (peaks in occurrence) and ‘repellors’ (lows in occurrence) that are automatically identified by the analysis by step size. Potential analysis of the simulated seagrass data (**A**) with mutualism in relation to seagrass mortality (*m*_*n*_). The attractors and repellors are identified per mortality *m*_*n*_ step size of 0.0025 day^−1^. (**B**) The potential analyses of the NDVI data, attractors and repellors are identified by the analysis per 0.1-m elevation interval adapted from^17^.

To examine the importance of feedbacks, and to compare model outcomes with observed dynamics of our study system, we used potential analysis, a statistical method to detect ‘basins of attraction’ along an environmental stress gradient. Potential analysis on the simulation data without mutualism yield only a single attractor with shoot densities close to zero (data not shown). This is because high-density seagrass beds are not stable in this model (see Fig. 1a). Very different dynamics are found in the simulation data from the model with mutualism. Potential analysis on the simulated data identifies a single stable attractor in when mortality is low (i.e. *m*_*n*_ < 0.023 day^−1^). Here, the mutualistic interaction keeps the system in a state with high shoot densities at carrying capacity (8000 ± 1200 shoots m^−2^ (mean ± SD)) (Fig. 3a). However, when *m*_*n*_ is increased beyond 0.023 day^−1^ and the system displays cycles (Fig. 3a), the potential analysis identifies two main attractors: one at high and one at very low shoot densities. The analysis identified two attractors here because the cycles stay in both a high and low state for a relatively long time compared to intermediate shoot densities due to the buffering effect of the mutualism (Fig. 1c).

Earlier published remote sensing analyses revealed a rather stable period in seagrass cover between 2007 and 2011, after which a sharp decline followed that appeared to be triggered by drought-induced desiccation stress and disruption of the mutualistic feedback^17^. Results from the potential analyses on the combined NDVI data of 2007, 2009, 2011 and 2013 are very similar to those from our model with the mutualism (Fig. 3a and b). At low mortality levels, potential analysis finds only a single attractor at high NDVI at low elevations where desiccation stress is absent or only minor. The analysis detects two attractors at intermediate elevations (−0.3 to 0 m MWL) (Fig. 3b). This indicates that areas with high seagrass cover started to shift to a degrading state in 2011, which is confirmed by a bimodal distribution in seagrass cover in that year (Supplementary Text 3). Finally, only a single attractor at low NDVI values is stable at high elevations where desiccation stress is typically high.

## Discussion

Mutualisms, due to their very nature, create a positive feedback between the species involved, and these feedbacks have been hypothesized to stabilize communities and ecosystems, especially if the mutualistic interaction involves a foundation species structuring the ecosystem^13,14^. In this study, we demonstrate how a mutualism-driven positive feedback buffers a negative feedback imposed by the foundation species upon itself, thereby stabilizing the ecosystem in a seagrass-dominated state. More specifically, simulations from our empirically calibrated model predict that in absence of the mutualism, anaerobic decomposition of organic matter accumulated by seagrass itself leads to damped oscillations due to excessive production of toxic sulfide. By contrast, in the presence of the seagrass-bivalve-bacteria mutualism, sulfide toxicity is alleviated because sulfide is consumed by the lucinid bivalve-bacteria consortium, thereby stabilizing the ecosystem under default conditions.

Our model analyses, however, also reveals an important inherent risk of mutualism-dependency: if the mutualism is weakened or overwhelmed due to enhanced environmental stress – as simulated by enhanced natural seagrass mortality here – the sulfide-buffering capacity of the mutualistic feedback can be exceeded, resulting in ecosystem collapse. Potential analyses on simulation results from the model with mutualism identified a single stable attractor at high shoot densities at default parameter settings. Here, any produced sulfide is immediately removed by the seagrass-lucinid mutualism. However, when mortality was enhanced beyond a certain threshold, a second attractor at low shoot densities was identified. In addition, the analysis predicts that seagrass shoot density for the highest attractor (i.e. seagrass-dominated state) gradually decreases with increasing mortality. These findings were similar to results we obtained from the potential analyses on the field data. In periods with low desiccation stress i.e. 2007 and 2009^17^, only a single attractor a high NDVI was stable and gradually decreased within increasing elevation. During the 2011-drought, however, a second low-NDVI attractor was identified, suggesting a feedback-mediated shift towards the degraded state observed in 2013^17^. Such ecosystem dynamics following gradual environmental change or perturbations of strong positive feedbacks have been described for a wide range of ecosystems^24,25^. For example, shallow lakes under excessive nutrient loading switch from clear to a turbid state^35^ and climate change-related shifts in plant-pollinator disruptions^28,36^; the expelling of zooxanthelae by corals leading to ‘coral bleaching events’^9,29^.

In many ecosystems, strong positive feedbacks can cause alternative states (i.e. bistability), implying that through gradually changing environmental conditions or a perturbation, a critical threshold can be crossed, causing a shift to an alternative state. If conditions subsequently improve, they have to progress beyond the point of collapse, before recovery to the initial state can take place, a phenomenon called hysteresis. In seagrass meadows, such hysteresis can for instance occur at high water column ammonia loading, as ammonia toxicity can only be alleviated through joint uptake and detoxification of ammonia by ample seagrass meadows of high density^37,38^. In our study system, however, the mutualistic feedback does not appear to lead to alternative stable states dynamics, but instead causes the occurrence of so-called “slow-fast” cycles under drought conditions. The dynamics of our model are similar to what was advanced as a potential explanation for cyclic shifts in shallow lakes where accumulation of phosphorus creates a “time bomb-effect” due to slow internal eutrophication^26^. In our system, the mutualism initially buffers sulfide production, but excessive organic matter accumulation (the “time bomb”) by seagrass at higher background mortality (*m*_*n*_) causes sulfide production to gradually increase and eventually outpace and overwhelm sulfide consumption by the mutualism, causing an abrupt shift to a bare state. Following the shift, however, organic matter is slowly exported from the system again, by erosion (the seagrass does not retain it anymore), followed by a period where seagrass can re-establish once organic matter and sulfide production drop below a certain threshold. This effectively causes slow-fast dynamics characterized by states that are persistent for long times, either seagrass or bare, with fast shifts (collapse and recovery) in between^26,34,39^.

Interestingly, our model predictions reveal that such slow-fast dynamics can create patterns (i.e. bimodality) that are similar or equal to the signature of alternative stable states in a potential analysis. So far, bimodality (multiple peaks in the frequency distribution) in potential analyses has mainly been attributed to the potential existence of multiple stable states^40–42^. Our results, however, suggest that caution is warranted when interpreting multimodality in potential analysis or bimodal frequency distributions, as multiple types of ecosystem dynamics, albeit all typically feedback-driven, may cause similar patterns in these analyses. As there are multiple examples of ecosystems with a potential for slow-fast dynamics^39,43–47^ our finding stresses the need for sufficient mechanistic insights when interpreting proxies or indicators for feedback-driven dynamics e.g. van der Heide *et al*., Weerman *et al*.^48–50^.

Our previous work suggests that the ecosystem collapse in our system was not a simple result of external environmental forcing but that (1) it was mediated by internal feedbacks, and (2) breakdown of the mutualistic feedback was as an important contributor^17^. Yet, thus far it was unknown to what extent the mutualism controls ecosystem stability, and whether its disruption may indeed cause the observed collapse. Here, we show that the seagrass-lucinid mutualism can indeed be vital in mediating both seagrass productivity and ecosystem stability^2,17^. Yet, even though a meta-analysis indicates that lucinids are present in 87% of all seagrass meadows worldwide^2^, it is currently unknown how the strength and the relative importance of the mutualism depend on abiotic conditions. As anaerobic degradation and related sulfide production are strongly temperature dependent, it is possible that mutualism strength will also depend on temperature. This appears to be supported by the lower proportion of studies that found seagrass-lucinid associations in subtropical (90%) and temperate seagrass beds (56%) compared to tropical meadows (97%)^2^. In addition, species-specific sulphide tolerance and sediment conditions such as organic matter and iron content that affect sulfide production, likely also affect mutualism dependency and thus potentially overall ecosystem dynamics^33^.

As current global climatic changes lead to increased average temperatures as well as an increase in the number of extreme events, it seems likely that stress events will become a more common phenomenon^51^. This is especially the case in the tropics where high temperatures in combination with strong winds can cause desiccation events, particularly during neap tides with prolonged low tide exposure^17,52–54^. Apart from temperature increase, however, there are also many other human impacts (e.g. eutrophication, siltation events) that threaten seagrass meadows and the mutualistic interactions that support it^55^. Many other coastal ecosystems with mutualism-dependent foundation species, such as coral reefs, sea- grass meadows, kelp forest, salt marshes, and mangroves, have also declined dramatically due to global change (e.g., global warming, eutrophication, and overfishing) over recent decades. Therefore, we suggest that a more mechanistic insight into these interactions and the resulting ecosystem dynamics is required and recommend that mutualisms involving foundation species should be considered as potential conservation and restoration targets.

## Methods

### Study system

This study was carried out in the intertidal area of Parc National du Banc d’ Arguin (PNBA) in Mauritania (19°52.42′N, 16°18.50′W). The area covers around 500 km^2^ of mudflat dominated by seagrass *Zostera noltii*^56^. Here, high densities (over 3700 ind. m^−2^) of the lucinid bivalve *Loripes lucinalis* inhabit silty, organic matter-rich sediment (up to 1-m thick) accumulated between the seagrass roots^2,57^.

### Model description

We developed a minimal differential equation model to investigate the importance of the mutualistic feedback between *Zostera* and *Loripes*, and the potential consequences of enhanced environmental stress for ecosystem stability. The changes in seagrass biomass are described as follows:

1 $$\frac{dZ}{dt}=r(1-\frac{Z}{{Z}_{max}})Z-f(mZ)$$

where *Z* is the seagrass shoot density (shoots m^−2^), *r* is the seagrass maximum net growth rate (day^−1^), *Z*_*max*_ is the carrying capacity (shoots m^−2^), and *f*(*mZ*) is a function describing seagrass mortality:

2 $$f(mZ)={m}_{s}\cdot \,fS\cdot Z+{m}_{n}\cdot Z$$

with *m*_*s*_ as the maximum mortality rate (day^−1^) due to sulfide toxicity and *m*_*n*_ as the natural seagrass mortality (day^−1^). The relative effect of the pore water sulphide concentration on toxicity-driven mortality is described by function *fS*:

3 $$\begin{array}{ll}fS=0\, & if\,S < {S}_{min}\\ fS=\frac{S-{S}_{min}}{{S}_{max}-{S}_{min}} & if\,S > {S}_{min}\,\& \,S < {S}_{max}\\ fS=1 & if\,S > {S}_{max}\end{array}$$

where *S* is the sulfide concentration (µmol L^−1^), *S*_*min*_ is the minimum sulfide concentration where toxicity occurs, and *S*_*max*_ is the concentration where toxicity is maximal. Here, we used a linear approximation of a toxicity curve where mortality gradually increases between *S*_*min*_ and *S*_*max*_ and is maximum above the latter^37,58^. Changes in sediment pore water sulfide concentrations are described according to the following differential equation:

4 $$\frac{dS}{dt}={C}_{om}\cdot OM-{C}_{s}\cdot L\cdot S-{e}_{s}\cdot S$$

*OM* describes the amount of sediment organic matter (%), *C*_*om*_ is a conversion factor (µmol L^−1^ %^−1^ day^−1^) relating organic matter decay to sulfide production, *C*_*s*_ is a conversion factor (m^2^ ind.^−1^ day^−1^) relating sulfide loss to consumption by *L Loripes* (ind. m^−2^), and *e*_*s*_ (day^−1^) describes loss of *S* due to chemical oxidation by oxygen diffusing into the sediment and diffusion of sulfide to the water layer^33^. For simplicity, we assume that organic matter finds its origin mainly from seagrass detritus in our pristine system and its dynamics are described by a third differential equation:

5 $$\frac{dOM}{dt}={C}_{z}\cdot f(mZ)-{e}_{m}\cdot OM$$

where *C*_*z*_ is the conversion factor (% m^2^ shoots^−1^) to convert dead seagrass biomass (sh m^−2^) to % and *e*_*m*_ (day^−1^) is the loss of organic matter due to decomposition and export. Finally we assume that *Loripes* is linearly dependent on seagrass biomass, as in addition to its dependency on sulphide, it depends directly on oxygen losses from seagrass roots, therefore follows a strong positive relation to seagrass density in the field^2^. Therefore, *Loripes* bivalve growth is described by:

6 $$\frac{dL}{dt}={r}_{L}(\frac{Z}{{Z}_{max}})(1-\frac{L}{{L}_{max}})-{m}_{L}\cdot L$$

where *L* is the *Loripes* density (ind. m^−2^) that is linearly dependent on seagrass biomass (*Z*), *r*_*L*_ is the growth rate (ind. m^−2^ day^−1^), *L*_*max*_ is the carrying capacity for *Loripes*, and *m*_*L*_ the natural mortality of *Loripes* (day^−1^). Note that *Loripes* spawns in the water column and growth rate is independent from the number of *Loripes* already present (see for example van der Meer *et al*.^59^).

### Model analysis

We first plotted model time plots at default settings (Table 1) without $(\frac{{\rm{dL}}}{{\rm{dt}}}=0)$ and with the mutualistic feedback, respectively. Next, we performed a bifurcation analysis in which we analysed the stability of the model for both scenarios over a wide range of settings of the parameter *m*_*n*_, which we used as a proxy for desiccation stress. This parameter was increased from 0 to 0.35 day^−1^ in 150 steps, and the analysis was also performed backwards to detect possible alternative equilibria in the model. Finally, we performed a sensitivity analysis on all model parameters at otherwise default settings, in which we determined the threshold value at which cycles occur (method based on van Nes *et al*.^26^). Model analysis was conducted with GRIND for MATLAB.

### Preparing field data for comparative analyses with the model

We calculated the Normalized Difference Vegetation Index (NDVI) as a proxy for seagrass cover to empirically study seagrass distribution over the mudflat elevation gradient at Banc d’Arguin. NDVI was calculated from Landsat 5 and 8 satellite images taken at low tide at the end of summer in the warmest months (Augustus-October) in 2007, 2009, 2011 and 2013 (Supplementary Texts 3 and 4). NDVI was calculated from the near-infrared (NIR) and red (RED) spectral bands: (NIR − RED)/(NIR + RED). To be able to combine and compare NDVI images, they were first standardized them by converting the digital numbers of the spectral bands to top-of-atmosphere reflectance. Next, we empirically cross-calibrated reflectance (2009 Landsat image served as baseline), using a linear model fitted to random common ground targets with low and high reflectance (ocean and desert respectively)^17,60,61^.

As a next step, we constructed a digital elevation model (DEM) dataset from Landsat images taken at varying tidal levels (Supplementary Text 4). We manually derived the contour lines of the water edge from a range of false colour image composites using Landsat SWIR, NIR and Green bands and combined these images into a single map. Different scenes that were taken in different phases of the tide were used, to find contour lines of the mudflats. Absolute elevation was assigned to the contour lines by determining the elevation of each contour line relative to mean sea level using real-time kinematic differential GPS in the field using a Trimble R8 GNSS. Finally, the DEM was created within ArcGIS 10 using 3D analyst by inverse-distance interpolation of the contour lines.

### Comparing model and field dynamics

To examine the role of the mutualistic feedback on ecosystem dynamics and compare model and field results, we analysed whether both the real and model system respond either gradually to increased environmental stress or display sudden shifts. If seagrass changes gradually in response to enhanced desiccation stress over the elevation gradient, the frequency distribution of seagrass cover should be unimodal, whereas feedback-mediated shifts between two states would typically result in a bimodal distribution frequency^17,41,62^. To examine the importance of possible feedbacks in our study system, we used potential analysis, a statistical method to detect alternative states or ‘basins of attraction’ along an environmental stress gradient^41,63,64^. Potential analysis is based on the fact that, if distinct ecosystem states exist due to the presence of strong internal feedbacks, transitions between these feedback-stabilized states or ‘attractors’ will occur rapidly because intermediate states are inherently unstable. Note that we do not necessarily define attractors as ‘classic’ alternative stables states here because there may be other, feedback-mediated dynamics (e.g. slow-fast cycles) that can yield similar results in the potential analysis (see results and discussion). By applying the potential analysis to both model and field data, we link our simulation results to the field situation and investigate whether the mutualism is indeed an important driver of seagrass dynamics in our study system.

Model data was prepared by simulating seagrass cover (*Z*_*max*_) simulated over 61 steps (step size: 0.0025 day^−1^) of mortality *m*_*n*_ (0–0.15). After stabilization of 200 years, the model was ran for another 50 years with the model state being saved at the end of each year. To obtain simulation data for empirically relevant stochastic noise, 625 models were run in parallel with varying levels of carrying capacity *Z*_*max*_ obtained from a stochastic noise function with a variance (*σ* = 0.15) that was estimated from the NDVI data.

Field data was prepared by calibrating all 17672 NDVI values of each map for every year (2007, 2009, 2011 and 2013) allocated in 9 elevation classes of 0.1 m from −0.6 to 0.3 meter relative to mean sea level, based on the digital elevation map (DEM). Within each elevation class NDVI values were divided in bins of size h = 1.06 × s × n^−1/5^ (s = standard deviation of *z* and n = number of observations/pixels) to get sufficient observations per bin to estimate the probability density function per elevation class. Data was analysed in a potential analysis with all years combined.

Following e.g. Livina and Lenton^65^ we assumed that the variation in the simulated and observed seagrass cover are the result of underlying feedbacks and stochastic processes, which in general terms can be described as: *dz* = −*U*′(*z*)*dt* + *σ dW*. The first term describes the deterministic dynamics of the system, in which *U*(*z*) is the potential (attractor) as the result of the feedback in the system. The second term describes the stochastic component, in which *σ* is the noise level and *dW* a Gaussian noise term. Here, the variable *z* denotes simulated seagrass biomass or NDVI. As we are interested in feedback-mediated bimodality in *z* the probability density function *P*_*d*_ was estimated for each elevation class using a standard Gaussian kernel estimator (with *ksdensity* function in MATLAB). The bimodality in the potential function *U* = −*log*(*P*_*d*_) in a mortality or elevation class is an indication of multiple states and the local minima and maxima of *U* correspond to stable and unstable attractors, respectively^62,64^. The low- and high frequencies in seagrass biomass or NDVI were identified in automated way with the *peaksearch* function in MATLAB.

## Electronic supplementary material

Electronic supplementary materials

Supplementary Dataset 1

## Data Availability

The datasets analysed during this study are included as Supplementary information files.
