# Supplementary material for: A facultative mutualistic feedback enhances the stability of tropical intertidal seagrass beds
Source: Sci Rep. 2018 Aug 28;8:12988. doi: 10.1038/s41598-018-31060-x (PMC6113270; doi:10.1038/s41598-018-31060-x)
Supplement: Supplementary file 1 — Electronic supplementary materials [file 41598_2018_31060_MOESM1_ESM.pdf]

Electronic supplementary materials for:

A facultative mutualistic feedback enhances the stability of tropical intertidal seagrass beds

Jimmy de Fouw[1,2*], Tjisse van der Heide[1,2,3], Jim van Belzen[4,5], Laura L. Govers[1,3] , Mohammed Ahmed Sidi Cheikh[3], Han Olff[3], Johan van de Koppel[4] and Jan A. van Gils[2]

[1]Department of Aquatic Ecology and Environmental Biology, Institute for Water and Wetland Research, Radboud University Nijmegen, Heyendaalseweg 135, 6525 AJ Nijmegen, The Netherlands

[2]Department of Coastal Systems, NIOZ Royal Netherlands Institute for Sea Research, and Utrecht University, P.O. Box 59, 1790 AB Den Burg (Texel), The Netherlands

[3]Groningen Institute for Evolutionary Life Sciences (GELIFES), University of Groningen, P.O. Box 11103, 9700 CC Groningen, The Netherlands

[4]Department of Estuarine and Delta Systems, NIOZ Royal Netherlands Institute for Sea Research, and Utrecht University, P.O. Box 140, 4400 CA Yerseke, The Netherlands

[5] Ecosystem Management Research Group, Department of Biology, University of Antwerp, Universiteitsplein 1, 2610 Wilrijk, Belgium

* Author for correspondence e-mail: j.deFouw@science.ru.nl

This file includes:

Supplementary text 1: *Supplementary text on model parameter estimation*

Supplementary table 2: *Sensitivity analysis of model parameters*

Supplementary text 3: *Results of potential analysis and bimodality test of NDVI data over the years (2007-2013)*

Supplementary text 4: *Landsat images used for elevation map and calculation of NDVI*

Supplementary text 1: model parameter estimation

Derivation of maximum seagrass mortality due to sulfide toxicity

We derived the maximum seagrass sulfide mortality m_s using a modified version of equation 1 in which we set the Hill-response curve to 1 (see main text). We used default values derived from the literature for all other parameters (table 1, see main text). Next, m_s was estimated at 0.5 day^{-1} where the maximum seagrass mortality was reduced by 80% after 6 days (Holmer and Bondgaard, 2001).

Estimation of conversion factor C_{om} relating percentage organic matter to sulfide production

Method – To investigate sediment pore water sulfide production in seagrass meadows in our study system, we conducted a laboratory experiment in our field station at Banc d'Arguin. We measured pore water sulfide production by filling 500 ml bottles anaerobically collected sediment (including seagrass detritus) from a healthy seagrass meadow (6770 shoots m^{-2} and 309.5 dry mass gram m^{-2}) (J. de Fouw, L. L. Govers and T. van der Heide unpub. data). Sediment was extracted from the upper 5 cm of the seagrass sediment layer in the field by using a Teflon tube (5 mm inner diameter) connected to vacuum 500 ml bottles and connected to a vacuum pump. In the laboratory, bottles (n=7) were wrapped in aluminium foil and incubated in water for three days at an average temperature of 26.8 C°. Pore water sulfide concentrations were measured daily by collecting pore water samples in 60-ml vacuumed syringes connected to 5-cm rhizon samplers (Eijkelkamp Agrisearch Equipment, Giesbeek, the Netherlands) (van der Heide et al., 2012b) that had been inserted into the bottles through an airtight rubber seal. The total dissolved sulfide concentration in the pore water was measured immediately after sampling, in a mixture of 50% sample and 50% Sulfide Anti-Oxidation Buffer (SAOB) using an ion-specific silver-sulfide electrode (Lamers et al., 1998). In addition, we measured sediment organic matter in the upper 5 cm of the sediment in the field. Sediment samples were taken with a small tube (10 ml), weighed immediately after collection and frozen (-10°C). Samples were freeze-dried in the laboratory, after which we determined organic matter as loss on ignition (LOI; 5 hrs at 560°C).

Result – Based on the organic matter content in the 10 ml tube we calculated the organic matter content for the top 5 cm per m^{-2}. The tube contained 0.45 g ml^{-1} of sediment dry mass containing 11.9% organic matter (OM) (de Fouw et al., 2016), which is: 0.054 g organic

matter ml^{-1} (11.9% of 0.45). Hence, the top 5 cm of a healthy seagrass beds contains than 2696 g organic matter m^{-2} (0.054 × 50 L / 0.001 mL). In our model Z is expressed in seagrass densities in shoots m^{-2}, therefore we first calculated the equivalent amount of organic matter in to shoots. Organic matter is converted to shoots m^{-2}, by dividing 2696 by the biomass of a single shoot (one shoot: 0.045 g; J. de Fouw, L. L. Govers and T. van der Heide unpub. data). This comes down to an organic matter equivalent of 59000 dead shoots m^{-2}. In this rich organic matter sediment sulfide concentration increased on average with 686.6 μmol L^{-1} day^{-1} . Hence, the sulfide conversion factor C_{om} was estimated at 686.6/59000 = 0.01 μmol L^{-1} shoots day^{-1} m^{-2}.

Conversion factor C_s relating loss of sulfide by uptake

Method – To investigate the sulfide uptake by *Loripes*, we conducted an experiment in the field station at Banc d'Arguin. *Loripes* for the experiment were collected from the seagrass meadows at Abelgh Eiznaya (19°53.54′ N, 16°18.85′W). The lower 6-cm tall sections of 8 two-compartment PVC columns (diameter 8.4 cm) were filled with seawater (figure 2; after van der Heide et al., 2012b). These 330-ml sections contained an injection tube and were separated from their upper compartments through a porous 0.1-mm membrane. Upper compartments were filled with sieved (1-mm mesh) iron-free silver sand to prevent formation and precipitation of iron-sulfides. Next, we placed five *Loripes* (7.44 ± 0.58 mm (mean± SD) per column (900 ind. m^{-2}) were added on top of the sediment surface, which subsequently buried themselves into the sediment. The columns were placed in plastic basins (57.0×39.0× 30.0 cm) with seawater where water flow and oxygen saturation were maintained with a pump. Salinity and pH where kept constant by adding fresh water and seawater when needed (measured with a 556 Multi Parameter Sampler, Yellow Springs Instruments). Sulfide was added every third day by injecting a 100 umol L^{-1} Na2S solution (set at pH 7.5 with HCl) into the lower compartment, 500 and 1000 umol L^{-1} respectively (1.5 and 3.0 ml). Prior to injection, pore water was sampled from the lower compartment and measured following the procedure above (see *Estimation of C_{om}*).

Results – Sulphide loss due to diffusion and/or oxidation in the control columns was negligible during the short 3-day experimental period. Hence, sulfide uptake by *Loripes* C_s was estimated at -0.0027 m^2 day^{-1} ind^{-1} using a non-linear model: $S_{tx} = S_{t0} \times e^{Cs \times L \times day}$ (Figure 2). Here, L is the *Loripes* density (900 ind. m^{-2}), S_{t0} is the sulfide concentration after injection,

and S_{ti} is the sulfide concentration at day i - we tested 500 and 1000 µmol L^{-1} respectively (see Figure 2).

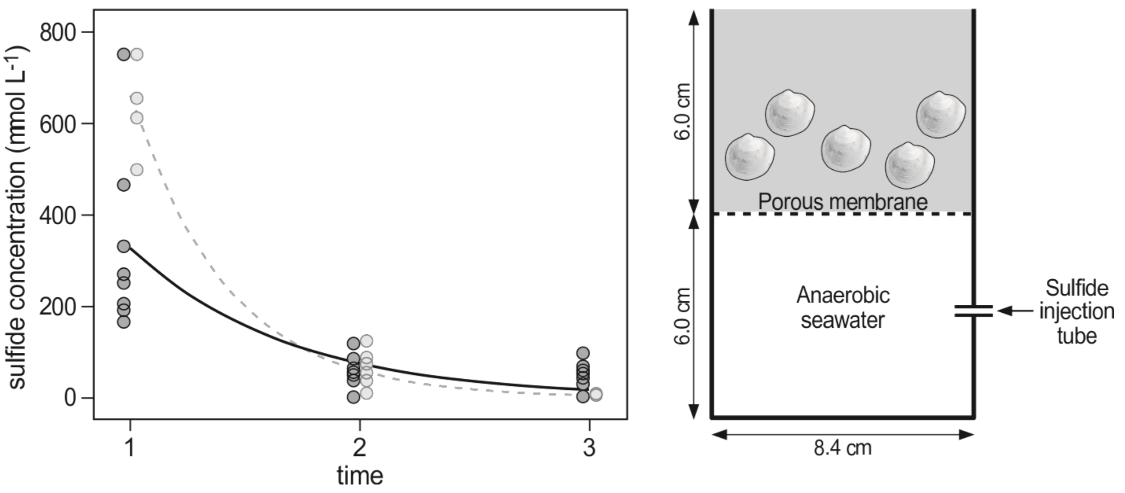

Figure S1. Sulfide loss by uptake of *Loripes* per day and schematic drawing of the setup of an experimental unit. Sulfide was injected every three days and allowed to diffuse from the lower compartment into the upper section through a 0.1-mm porous membrane. Parameter *Cs* was estimated based on injection of 500 and 1000 µmol L^{-1} sulfide at t=0, black line and blue line respectively.

Loss of sulfide e_s due to chemical oxidation and loss to the water layer

Apart from sulfide uptake by *Loripes* there is a constant loss of sulfide due to chemical oxidation and diffusion to the water layer. We derived this constant loss of sulfide using a modified version of equation 3 in which we assumed that there was no *Loripes* resulting in no sulfide uptake by *Loripes*: $\frac{dS}{dt} = C_{om} \cdot OM - e_s \cdot S$ where *S* is de maximum sulfide concentration (2000 µmol L^{-1}) in healthy seagrass with a sediment organic matter of 11.9% (see *Estimation of C_{om}*). Sulfide loss e_s was estimated at 0.29 day^{-1}.

Estimation of conversion factor C_z shoots to relating percentage organic matter

Organic matter *OM* is expressed in percentage therefore we need to convert *f(mZ),* the function describing seagrass mortality, to percentage with a conversion factor C_z. To calculate C_z we estimated the maximum organic matter content in healthy seagrass, by dividing 11.9%

organic matter by 59000 shoots m^{-2} (see details on numbers C_{om}) and was estimated at 2.017×10^{-6} µmol L^{-1} % $^{-1}$ day^{-1}.

Loss of organic matter e_m due decomposition and export

We assume a constant loss of organic matter e_m from the system due to decomposition and export. Although our model does not include a function of hydrodynamics which effects OM we implicitly include this by using field data. Therefore we used again a maximum sediment organic matter of 11.9% for a healthy seagrass bed (with an equivalent of 59000 shoots m^{-2}, see calculation for C_{om}) and using only natural seagrass mortality rate m_n as a source of input. Next, daily loss of organic matter e_m was derived by isolating equation 4 (see main text) and estimated to be 0.0009 day^{-1}.

Supplementary table 2: Sensitivity analysis of model parameters

Table S2 Sensitivity analysis of model parameters relative change and model dynamics. The sensitivity of the cycles is defined as the relative change and the threshold value of the parameters which is required for cyclic dynamics, or model collapse for m_s.

Parameters	Description (unit)	Default value	Relative change required for cycles	Threshold value of model dynamic change	Model dynamics
Z_{max}	maximum seagrass density (carrying capacity) (shoots m^{-2})	8000	313% increase	>25000	cycles
r	relative growth rate seagrass (day^{-1})	0.35		infinite	na
m_s	maximum seagrass mortality rate by sulfide (day^{-1})	0.5	99% reduction	<0.0065	collapse
S_{min}	Minimum sulfide concentration where toxicity occurs (µmol L^{-1})	200	69% reduction	<62	cycles
S_{max}	Sulfide concentration where the toxicity effect becomes maximal (µmol L^{-1})	1000		infinite	na
C_{om}	conversion factor relating % OM to sulfide (µmol L^{-1} % $^{-1}$ day^{-1})	49.57	323% increase	>160	cycles
C_s	conversion factor relating loss of sulfide by uptake (ind^{-1} day^{-1} m^{2})	0.0027	19% reduction	<0.0022	cycles
e_s	loss of S due to chemical oxidation and loss water layer (day^{-1})	0.29		infinite	cycles
C_z	Conversion factor for sh/m^2 to OM% (%shoots^{-1} m^{2})	2.02E-06	346535% increase	>0.0007	cycles
e_m	loss of OM due decomposition and export (day^{-1})	0.0009	67% reduction	<0.0003	cycles
r_L	relative growth rate *loripes* (ind day^{-1} m^{-2})	26	88% reduction	<3	cycles
L_{max}	maximum *loripes* density (carrying capacity) (ind m^{-2})	4,900	78% reduction	<1100	cycles
m_L	natural *loripes* mortality rate (day^{-1})	0.002	950% increase	>0.019	cycles

Supplementary text 3: Results of potential analysis and bimodality test of NDVI data over the years (2007-2013)

NDVI analysis on temporal dynamics and spatial heterogeneity of seagrass cover revealed a rather stable period in seagrass cover between 2007 and 2011 after which a 44% decline followed between 2011 and 2013 (Figure S2 A-D) which was found to be associated with drought-induced enhanced desiccation stress and disruption of the mutualistic feedback (de Fouw et al., 2016). As first indication for feedback-mediated dynamics we used the frequency distribution and tested on bimodality (*bimodalitytest*), using the software program R (R Development Core Team 2014, Holzmann and Vollmer 2008). The frequency distribution of NDVI showed a significant bimodal distribution for 2011 ($P < 0.001$), but this was not detected in 2007, 2009 and 2013 (P=0.5) (Figure S2 E-H). This bimodality suggests a feedback-mediated sudden shift rather than a gradual response. This was supported by the potential analysis of the NDVI data. Similar to the results from our model with mutualism at low mortality levels (i.e. where the system is stable) (see main text), analyses on maps of 2007 and 2009 revealed one point of attraction that decreased with increasing elevation, suggesting that desiccation was an important factor causing lower seagrass cover in areas of higher elevation and exposure times (Figure S2 I-J). During the drought event in 2011, the analysis identifies two attractors at intermediate elevations (-0.4 to 0 m MWL), indicating that areas with high seagrass cover shifted to a degrading state (Figure S2 K). Furthermore, the higher attractor decreases with increasing elevation, again indication that seagrass cover is related to desiccation. In 2013 most areas at intermediate elevations had transitioned from a high seagrass cover state to a degraded state as only the lower attractor was stable above -0.4 m MWL (Figure S2 L).

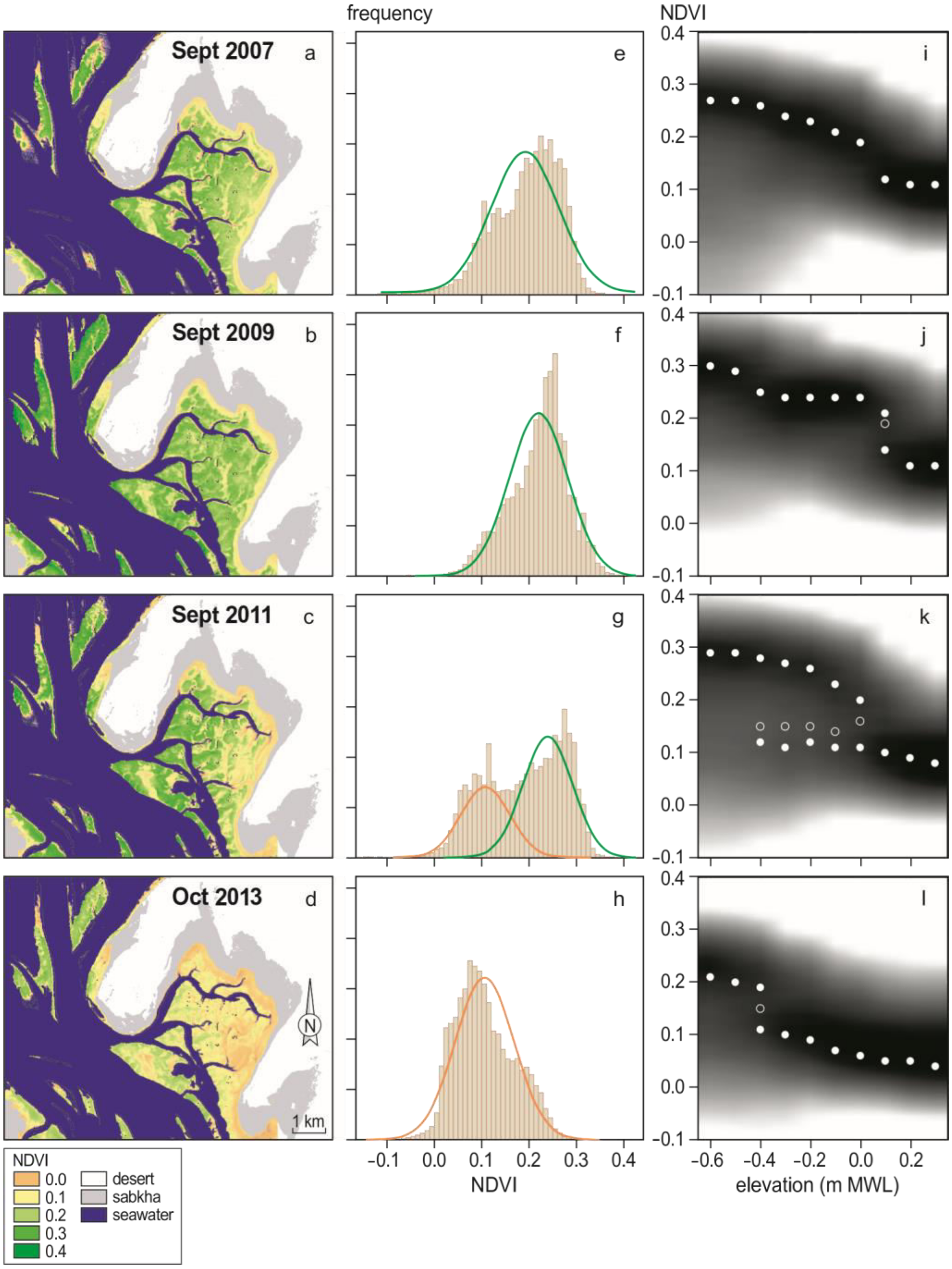

Figure S2. (A-D) NVDI in our study area (Banc d'Arguin, Mauritania) from 2007 to 2013 as calculated from Landsat imagery at low tide. (E-H) Frequency distribution of NDVI data and (I-L) the potential analyses of the NDVI data, dark and light shades depict NDVI ranges of high and low occurrence, respectively; closed and open markers depict 'attractors' (peaks in occurrence) and 'repellors' (lows in occurrence) that are automatically identified by the analysis per 0.1-m elevation interval. Figures A-D and I-L were adapted from (de Fouw et al. 2016).

Supplementary text 4: Landsat images used for calculation of NDVI and elevation map

Digital elevation map was based on 9 Landsat images with different tide levels (table S3). Contour lines of the water edge were manually derived from a false color image composite using Landsat SWIR, NIR and Green bands and combined into a new map

Table S3. Landsat images to construct the Elevation map.

Landsat image	sea level	time acquisition
1973-02-22 LS1MSS	-1.3	11:01:40
2001-12-18 LS7ETM	-1	11:20:55
2011-09-17 LS5TM	-0.95	11:20:31
2010-07-28 LS5TM	-0.58	11:22:29
2011-01-04 LS5TM	-0.3	11:21:58
2014-03-17 LC8	-0.4	11:32:31
2014-03-01 LC8	-0.2	11:32:40
2011-07-15 LS5TM	0.1	11:21:11
2011-06-13 LS5TM	0.2	11:21:24

Satellite images used for the analysis which were taken at low tide at the end of summer in the warmest months Suitable (i.e. regarding season, low tide, haziness) Landsat images used for the GIS analyses (table S4)

Table S4 Landsat images used for the GIS analyses to calculate NDVI.

Image	*date*	*time*	*Mapping*
Landsat 5	15-Sep-2007	11:18:58	NDVI
Landsat 5	11-Sep-2009	11:21:45	NDVI (baseline map)
Landsat 5	17-Sep-2011	11:20:31	NDVI
Landsat 8	24-Oct-2013	11:33:49	NDVI

References

de Fouw, J., L. L. Govers, J. Van Belzen, J. van de Koppel, W. Dorigo, M. J. A. Christianen, K. J. van der Reijden, M. van der Geest, T. Piersma, A. J. P. Smolders, H. Olff, L. P. M. Lamers, J. A. van Gils, and T. van der Heide. 2016. Drought, mutualism breakdown and landscape-scale degradation of seagrass beds. Current Biology **26**:1051–1056.

Holzmann, H., and S. Vollmer. 2008. A likelihood ratio test for bimodality in two-component mixtures with application to regional income distribution in the EU. Advances in Statistical Analysis **92**:57-69.

R Development Core Team. 2014. R: A language and environment for statistical computing. R Foundation for Statistical Computing, Vienna, Austria.
